# A high-throughput chemical screen identifies novel inhibitors and enhancers of anti-inflammatory functions of the glucocorticoid receptor

**DOI:** 10.1038/s41598-017-07565-2

**Published:** 2017-08-07

**Authors:** Xiaofeng Jiang, Amber Dahlin, Scott T. Weiss, Kelan Tantisira, Quan Lu

**Affiliations:** 1 Program in Molecular and Integrative Physiological Sciences, Departments of Environmental Health, and Genetics and Complex Diseases, Harvard T.H. Chan School of Public Health, Boston, Massachusetts USA; 2 Channing Division of Network Medicine, Brigham and Women’s Hospital, Harvard Medical School, Boston, Massachusetts USA

## Abstract

Glucocorticoids (GCs)—ligands of the glucocorticoid receptor (GR)—are widely used to treat inflammatory diseases, but suffer from significant side effects and poor responsiveness in certain patient populations. Identification of chemical GR modulators may provide insights into the regulatory mechanisms of anti-inflammatory functions of GR and help improve GC-based therapy. Here we report the development and application of a high-throughput screening to identify compounds that either enhance or suppress the anti-inflammatory effect of GR function. Using a cell-based GR activity assay that measures Dexamethasone (Dex)-mediated NF-κB repression, we have screened ~8,000 compounds and identified several compounds that suppressed GR activity, including multiple GSK3β inhibitors and anti-cancer agent camptothecin. Notably, we also identified two kinase IKK2 inhibitors, including TPCA-1, as GR enhancers that improve the anti-inflammatory effect of GR. In particular, TPCA-1 augmented the activity of Dex in NF-κB repression by attenuating GR down-regulation. Consistent with the observation, siRNA-mediated IKK2 knockdown decreased GR down-regulation and increased GR expression. Together, our results identified chemical compounds as novel modulators of GR and revealed an unexpected role for IKK2 in GR down-regulation. Furthermore, we have established a high-throughput screening platform for discovering GR-modulating compounds that may be repurposed to improve current GC-based therapies.

## Introduction

Inflammation underlies the pathogenesis of many lung diseases, including asthma and COPD. Consequently, a major goal of therapeutic intervention for these diseases is to reduce airway inflammation. Glucocorticoids (GCs), a major class of anti-inflammatory drugs, are widely used to treat both asthma and COPD^1, 2^. While generally effective and well-tolerated, GCs can cause significant side effects, including impaired growth in children, decreased bone density and osteoporosis, and glaucoma^3^. Moreover, a significant portion of the patient population does not respond well to GCs and many asthma patients with severe symptoms are resistant to GC-based therapies^4, 5^. Thus, there is an urgent need to improve GC-based therapy for patients.

GCs exert their anti-inflammatory effects through activating the glucocorticoid receptor (GR)^6, 7^. In the absence of GC binding, GR is inactive and resides in the cytoplasm in a complex with other proteins including chaperone proteins hsp90 and hsp70^8^. Upon GC binding, GR dissociates from the inhibitory complex and rapidly trans-locates to the nucleus^9–11^, where GC-bound GR acts as transcription factor to activate or suppress the expression of a large number of target genes^12^. GR activates gene expression by directly binding to specific DNA elements known as glucocorticoid response elements (GREs) in the promoter regions of target genes^13^. GR also suppresses gene expression by binding to negative GREs (nGREs) of target genes^14^. Remarkably, in addition to directly binding to chromosomal DNA to regulate gene expression, ligand-bound GR can also associate and interfere with the activity of other transcription factors such as NF-κB and STATs, resulting in a state of “tethered trans-repression” in which the expression of the gene targets of those transcription factors is reduced^15, 16^.

Whereas both transcriptional activation and tethered trans-repression contribute to the overall anti-inflammation action of GR, tethered trans-repression is thought to be the major mechanism by which GCs suppress airway inflammation and thereby relieve asthma symptoms^17^. Efforts to improve GC-based therapies have mostly focused on developing selective GR ligands that promote tethered trans-repression and attenuate transcriptional activation of pro-inflammatory genes^18, 19^. However, it remains uncertain whether such rational chemical design approaches will yield truly selective GR ligands that promote a desirable effect on transcriptional activity^20^. An alternative approach is to identify non-ligand modulators of GR, and several studies have identified small molecules that modulate different aspects of GR function, including ligand binding, GRE dependent transcriptional activation and repression^21–23^. In this study, we developed a specific tethered trans-repression based assay and report the identification and characterization of GR modulators in a high-throughput screen of ~8,000 bioactive compounds.

## Results

### Establishment of a lung epithelial cellular model to assay GC-mediated tethered transrepression

**Figure 1:**
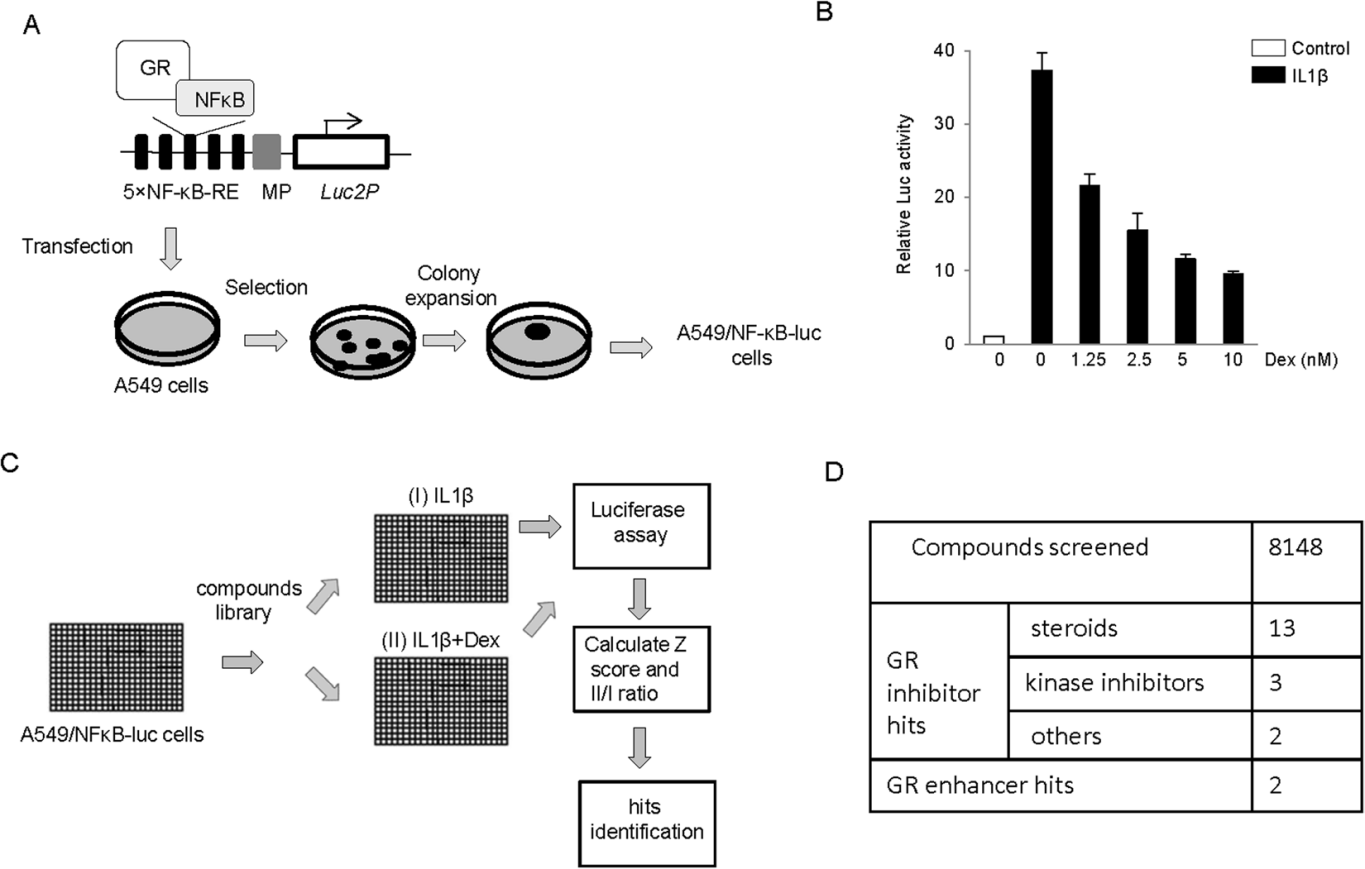
A high-through chemical screen to identify GR modulators. (**A**) Establishment of a stable reporter cell line A549/NF-κB-luc to assay tethered trans-repression of NF-κB activation by GR. A plasmid in which five tandem NF-κB responsive elements (5 × NF-κB-RE) was put upstream of mini promoter (MP) to control *Luc2P* expression was stably transfected into A549 cells. The individual colonies were selected, expanded and characterized. (**B**) A549/NF-κB-luc reporter cells were sensitive to IL-1β in NFκB activation and sensitive to Dex in NF-κB repression by GR. Cells were treated with 5 ng/mL IL-1β ± Dex as indicated for 18 h and Luciferase assay was performed. (**C**) Schematic representation of the chemical screen design. A549/NF-κB-luc reporter cells were seeded in 384 well plates, and library compounds were added to the cell plates. One hour later, 5 ng/mL IL-1β was added to assay I plates and 5 ng/mL IL-1β + 2.5 nM Dex was added to assay II plates. Luciferase assay was performed after 18 h treatment and MAD based Z scores for each well and ratio II/I of each tested compound were calculated. The inhibitor and enhancer hits were selected based on predefined criteria (see *Results*). (**D**) Summary of the GR inhibitors and enhancers identified in the screen.

Tethered trans-repression of GR on NF-κB is a major mechanism underlying the anti-inflammatory effect of GCs^24, 25^. We therefore first established a cell-based assay to quantify the effect of GCs in suppressing the transcriptional activity of NF-κB. To accomplish this, we first stably transfected the lung epithelial A549 cell line with a luciferase reporter, in which five tandem NF-κB responsive elements were placed upstream of a minimal promoter to drive luciferase expression (Fig. 1A). A cell line stably expressing the NF-κB luciferase reporter (A549/NF-κB-luc reporter) was selected and tested to determine the effect of GC on tethered trans-repression of NF-κB activation. As shown in Fig. 1B, treatment of the A549/NF-κB-luc reporter cells with IL1β, an inflammatory cytokine well known to activate NF-κB, increased the luciferase activity by ~36 fold. We then treated the A549/NF-κB-luc reporter cells with Dexamethasone (Dex), a synthetic GR agonist, at different concentrations in the presence of IL1β. Consistent with the anti-inflammatory effect of GCs, Dex reduced IL1β-induced increase in luciferase activity in a dose-dependent manner (Fig. 1B). These data indicate that A549/NF-κB-luc reporter cells exhibit GC-mediated tethered trans-repression of NF-κB.

### A high-throughput chemical screen identified compounds that modulate GR activity

Using the A549/NF-κB-luc reporter cells, we designed and optimized a two-assay-based high-throughput chemical screen to identify GR modulators (Fig. 1C). A549/NF-κB-luc reporter cells seeded in 384 well plates were first treated with compounds for1 hr and then treated with either 5 ng/mL IL-1β (Assay I), or with 5 ng/mL IL1β plus 2.5 nM Dex (Assay II) for 18 hr, followed by luciferase measurements (Fig. 1C). Assays I and II assessed NF-κB activation and Dex-mediated NF-κB repression, respectively. In particular, we chose to use 2.5 nM Dex that suppressed NF-κB activity by about half to allow for the identification of both inhibitors and enhancers of GR in the same screen. To assess the quality of our screen, we calculated the Z factor of the assays, which is widely used to assess the sensitivity and the data variation associated high-throughput screens^26^. Z factors were determined to be 0.69 and 0.64 for Assay I and II, respectively, indicating that the screen was sufficiently robust and sensitive to detect the effects of compounds on NF-κB activation and Dex-mediated NF-κB repression.

To select screen hits, we calculated the median absolute deviation (MAD)-based Z score (Z’)^27^ of compounds in assay II (Z’II), the normalized Luc readings in assay I *vs*. DMSO control (DMSO%I), and most importantly, the ratio of luciferase activity in assay II *vs*. that in assay I (II/I ratio). The ratio is assessed to minimize the direct effect of compounds on NF-κB activation and thus helps identify more GR specific modulators. For compounds with Z’II > 3, those with DMSO%I value in the range of 0.8-1 and II/I ratio being 50% higher than DMSO control were considered to be GR-specific inhibitors. The criteria of DMSO% I value in the range of 0.8-1 is used to test the effect of compounds on NF-κB activation and filter out NF-κB activators which are less interesting than GR inhibitors in the screen. Among the compounds with Z’II < −3, the GR specific activator was defined to be with II/I ratio being 20% lower than DMSO control.

We used the above high-throughput assay to screen 8148 bioactive compounds. They include 640 FDA approved drugs, 731 molecules from NIH clinical trial collections, 899 synthesized kinase inhibitors and a large number of commercial bioactive compounds. These compounds are selected to maximize chemical structure and biological pathway diversity. In total, we identified 18 GR inhibitors and 2 GR enhancers (Fig. 1D and Supplementary Table S1).

### Validation of GR inhibitors

**Figure 2:**
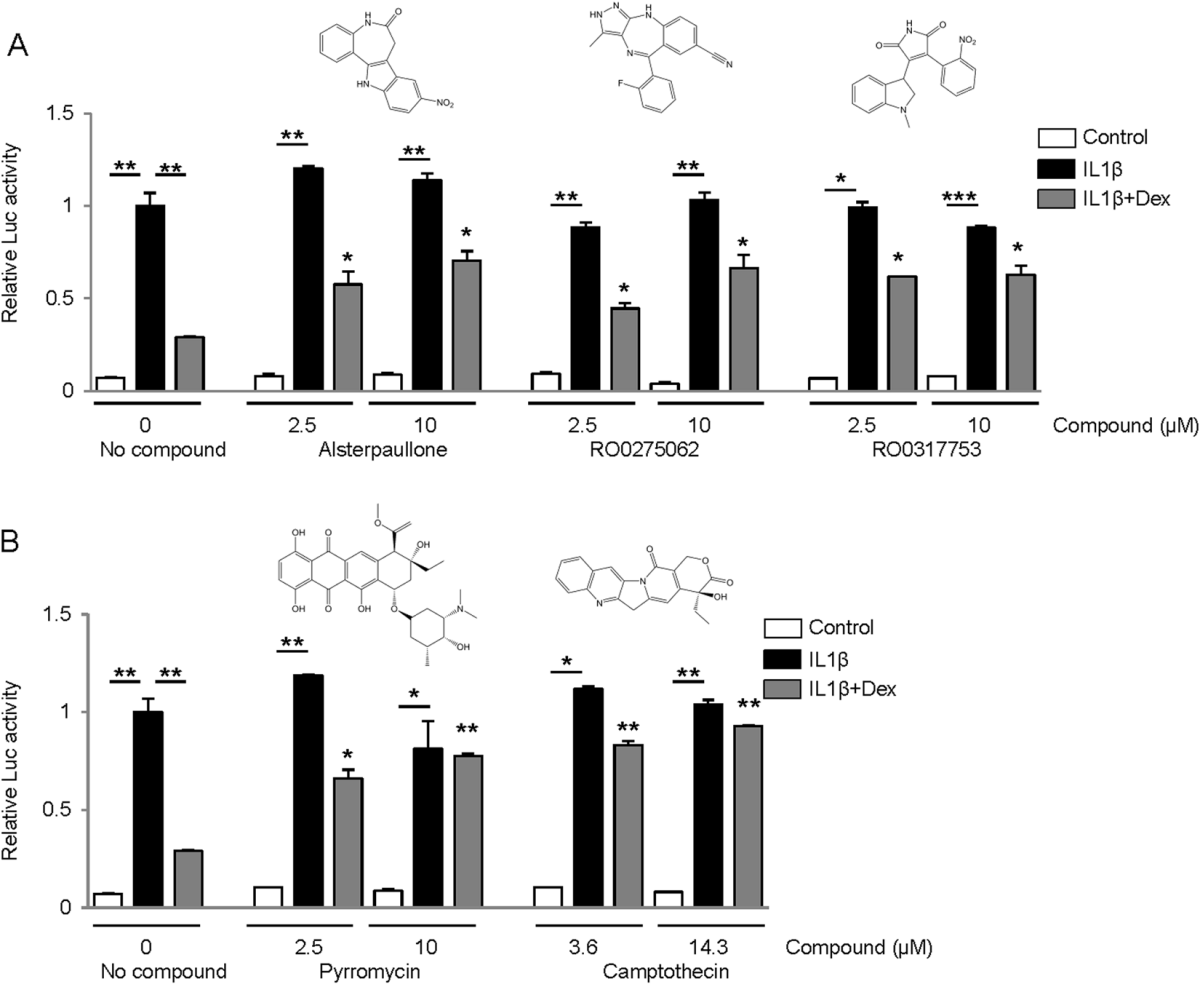
Validation of GR inhibitor hits. A549/NF-κB-luc reporter cells were treated with 5 ng/mL IL-1β ± 10 nM Dex and a pan-kinase inhibitor or kinase GSK3β inhibitors (**A**) or other compounds (**B**) as indicated. Luciferase assays were performed after 18 h treatment. The luciferase activity in control cells with 5 ng/mL IL-1β treatment was normalized to 1. The luciferase activity of cells treated with IL-1β + Dex and hits was calculated and compared to that of cells treated with IL-1β + Dex. *P < 0.05. **P < 0.01, ***P < 0.005.

Among the identified GR inhibitors were 13 steroids and 5 non-steroid compounds. While identification of the steroids provided validation for our screen, steroids also compete with GC in GR binding and inhibit GC functions^21^. Therefore, we did not further characterize the steroid compounds, but instead focused on validation of the non-steroid hits (Fig. 1D). Among the non-steroid GR inhibitors, we identified a pan-kinase inhibitor alsterpaullone and two inhibitors of glycogen synthase kinase 3β (GSK3β) (RO0275062 and RO0317753). Phosphorylation plays an important role in GR activation and function^28^. Treatment of the pan-kinase inhibitor alsterpaullone, as expected, significantly attenuated Dex-mediated suppression of NF-κB activity. Interestingly, it had a comparatively smaller effect on IL-1β-induced NF-κB activation (Fig. 2A). The GSK3β inhibitors similarly inhibited Dex-mediated suppression of NF-κB activity (Fig. 2A), a finding that is consistent with the reported implication of GSK3β in regulating GR function^29, 30^. The remaining GR inhibitors identified from the screen were two DNA intercalating anti-cancer compounds (pyrromycin and camptothecin). Pyrromycin is a monosaccharide anthracycline^31^, and camptothecin is a DNA topoisomerase I inhibitor, whose analogues have been used in cancer therapy^32^. Both compounds exhibited reproducible inhibition on NF-κB suppression by Dex (Fig. 2B).

### Identification and validation of IKK2 inhibitors as GR enhancers

**Figure 3:**
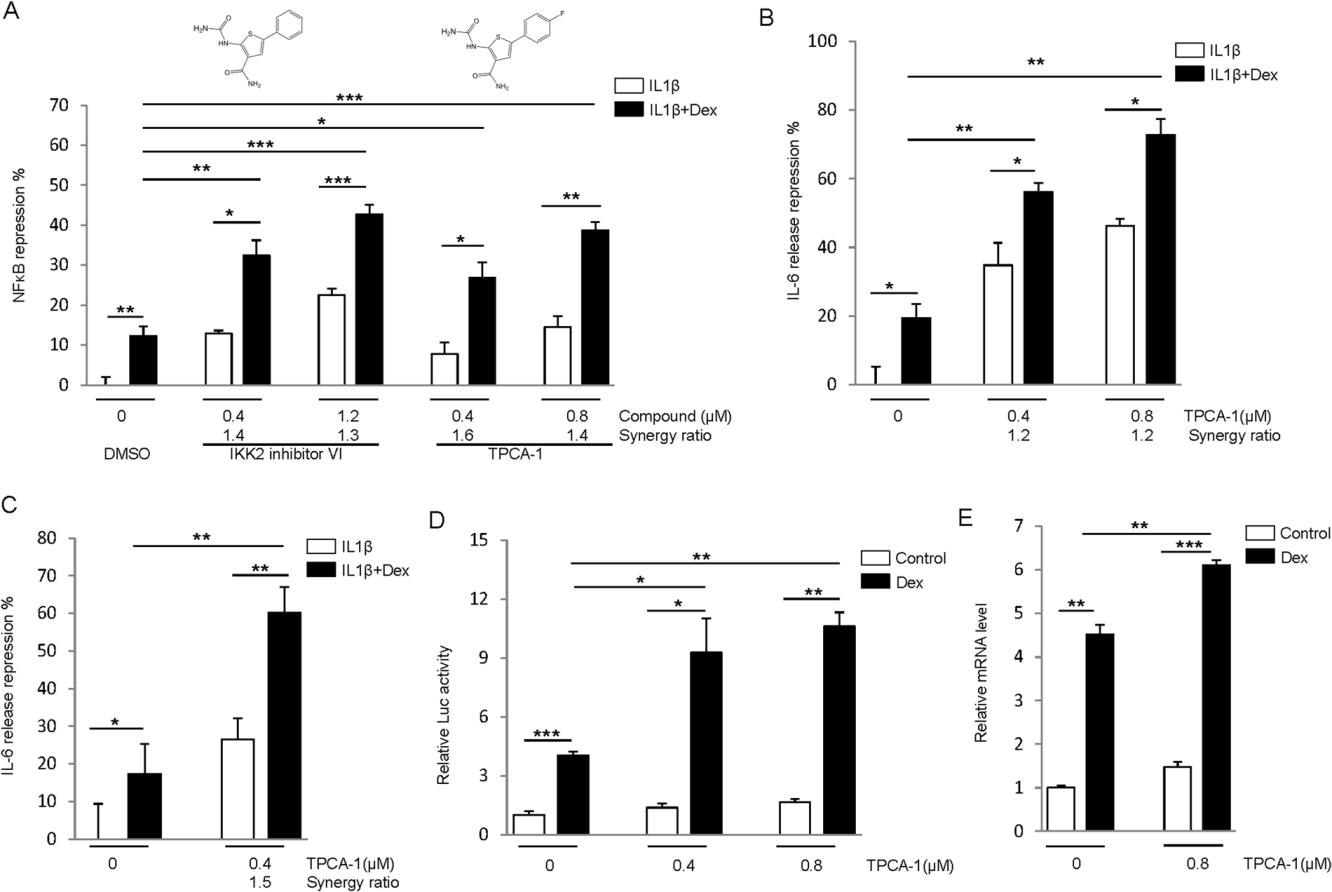
IKK2 inhibitors enhance GR activity. (**A**) IKK2 inhibitors enhance NF-κB repression by GR. The reporter cells were treated with 5 ng/mL IL-1β ± 0.5 nM Dex, and inhibitors as indicated for 18 h. Luciferase assays were performed, and NF-κB repression was calculated relative to the cells treated with IL1β. The synergy ratio is shown. (**B,C**) TPCA-1 enhances IL-6 release inhibition by GR. A549 cells (**B**) or human airway smooth muscle cells (**C**) were treated as in (**A**), and the level of IL-6 in medium was assayed by ELISA. (**D**) IKK2 inhibitor enhances trans-activation by GR. A549 cells were transfected with pGILZ-luc plasmid for 48 h, and then2.5 nM Dex and TPCA-1 were added as indicated. After 18 h treatment, luciferase assays were performed. **(E)** A549 cells were treated with 0.8 μM TPCA-1 and 2.5 nM Dex for 18 h, and qPCR was used to measure GILZ expression. The relative mRNA levels are shown. *P < 0.05. **P < 0.01, ***P < 0.005.

While GR inhibitors are mechanistically interesting for GR regulation, GR activators have the greatest potential to directly improve GR function and GC-based therapy. Our screen identified two compounds, TPCA-1, and IKK2 inhibitor VI, as potential enhancers of GR (Fig. 3A). Notably, TPCA-1 and IKK2 inhibitor VI are structurally similar and both are inhibitors of IKK2, a kinase essential for NF-κB activation^33, 34^. Consistent with the role of IKK2 in NF-κB activation, both TPCA-1 and IKK2 inhibitor VI slightly (~5–10%) inhibited IL-1β induced NF-κB activation (Fig. 3A). Both compounds also significantly increased NF-κB suppression by Dex from ~10% to up to 40% (Fig. 3A). The synergy ratio, a function that reveals the cooperative effects of the two treatments^35^, was greater than 1 for all combinations. These results confirmed that TPCA-1 and IKK2 inhibitor VI enhance the GR function in NF-κB repression and validated them as enhancers of GR activity.

TPCA-1 exhibited better GR-enhancing activity than inhibitor VI. Furthermore, it has been extensively studied as an anti-inflammation agent *in vitro* and *in vivo*, and even proposed for a clinical trial to treat inflammation diseases^36^. To further confirm the effect of TPCA-1, we tested the effect of the GR antagonist RU486, which, as expected, reduced the inhibitory effect of Dex on NFkB (Supplementary Fig. 4). Importantly, RU486 significantly attenuated the synergistic effect between Dex and TPCA-1 (Supplementary Fig. 4). RU486 did not affect the TPCA-1 effect on NFkB in the absence of Dex treatment. This result indicates that the effect of TPCA-1 on Dex-mediated NFkB suppression is dependent on GR. We then examined the effect of the compound on the production of cytokines such as interleukin 6 (IL-6) controlled by NF-κB activation. IL1β induced dramatic IL-6 release in A549 cells (Supplementary Figure S1B), and as expected, treatment of DEX attenuated IL6 level in the cells (Fig. 3B). Importantly, treatment of TPCA-1 further enhanced the inhibitory effects of DEX on IL6 production with a synergy ration of 1.2 (Fig. 3B). The synergistic effect of TPCA-1 and DEX on IL6 production was similarly observed in the primary human airway smooth muscle cells (Fig. 3C). Together these results indicate that TPCA-1 enhances GR-mediated repression of NF-κB signaling and attenuates pro-inflammatory cytokine production.

To examine the effects of TPCA-1 on trans-activation (another important function of GR), we transiently transfected A549 cells with plasmid pGILZ-luc in which the regulatory element of GR trans-activation target gene GILZ (glucocorticoid-induced leucine zipper)^37^ controls luciferase expression. While TPCA-1 itself had little effect on luciferase activity, the compound increased luciferase activity upon the treatment of Dex over different concentrations (Fig. 3D). This result suggests that TPCA-1 enhances the trans-activation function of GR. To further test whether TPCA-1 enhances GR function under physiological conditions, we used qPCR to measure the expression of endogenous GILZ. While the compound did not alter the basal level of GILZ expression, it moderately increased Dex induced expression of GILZ (Fig. 3E). Together, these results indicate that TPCA-1 enhances both tethered repression and trans-activation activities of GR.

### IKK2 inhibition attenuates GR down-regulation

**Figure 4:**
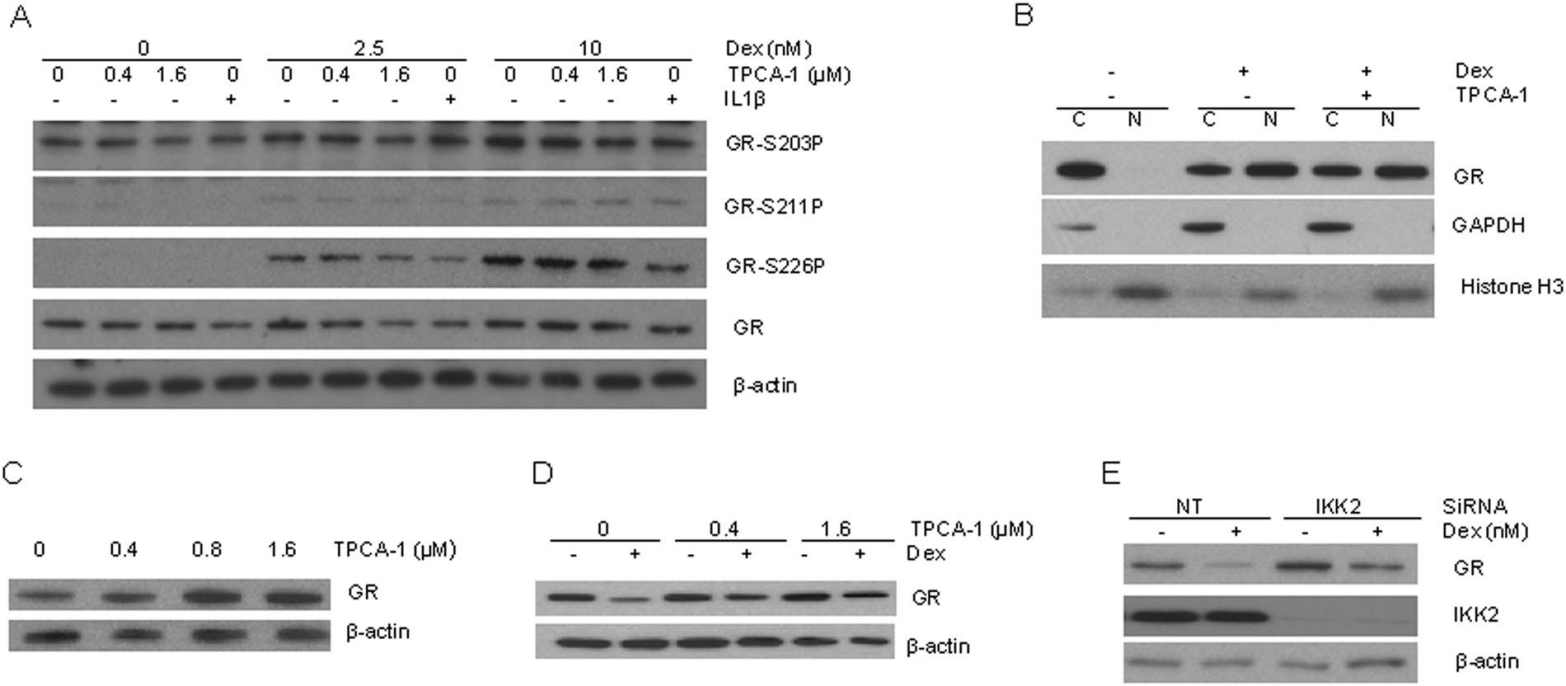
TPCA-1 increases GR level and impairs GR down-regulation. (**A**) TPCA-1 does not change GR phosphorylation level. A549 cells were treated for 30 min with Dex and TPCA-1 as indicated. Immunoblotting analysis was performed to measure GR phosphorylation level. (**B**) TPCA-1 does not alter Dex induced nuclear translocation of GR. A549 cells were treated as in (**A**). The cytoplasmic (**C**) and nuclear fractions (N) were prepared and analyzed using immune- blotting to assay GR level. GAPDH and histone H3 were applied as internal standards for cytoplasmic and nuclear fractions, respectively. (**C**) TPCA-1 increases GR level. A549 cells were treated with different concentrations of TPCA-1 for 48 h and crude cell extract was immune-blotted to measure GR level. (**D**) TPCA-1 impairs Dex induced GR down-regulation. A549 cells were treated with Dex and TPCA-1 as indicated, and GR level was analyzed. (**E**) IKK2 modulates GR level and down-regulation. A549 cells were transfected with Non-targeting (NT) or IKK2 siRNA for 48 h, and then treated with Dex for additional 48 h. Immuno-blotting was used to analyze GR and IKK2 levels. Full-length blots are presented in Supplementary Figure S4.

To explore the mechanism by which TPCA-1 potentiates GR activity, we examined the effects of the compound on key steps of the GR signaling pathway, including GR phosphorylation and translocation^28^. Dex induced significant GR phosphorylation on multiple serine residues (203, 211, and 226) (Fig. 4A). However, TPCA-1 treatment did not alter the levels of Dex induced GR phosphorylation at any of the serine residues (Fig. 4A). We then tested whether TPCA-1 interferes with GR translocation to the nucleus using cell fractionation. While GR is normally localized in the cytoplasm, upon Dex stimulation, a significant amount of GR was trans-located to the nucleus (Fig. 4B). The ratio of cytoplasmic and nuclear GR remained the similar level upon the additional treatment of TPCA-1. Together, these results indicate that TPCA-1 does not affect either GR phosphorylation or nuclear translocation, two essential steps in GR activation.

IKK2 has been reported to promote the stability of some proteins such as p53^38^ and Aurora A^39^. We thus examined if TPCA-1 alters GR protein level. As shown in Fig. 4C, TPCA-1 treatment led to a moderate increase in GR protein level in a dose-dependent manner. The mRNA level of GR gene was not affected (Supplementary Figure S2), suggesting that TPCA-1 affects GR protein stability. As GR protein expression is known to be down-regulated upon GC stimulation^40^, we therefore tested whether TPCA-1 affects GR down-regulation. As shown in Fig. 4D, while Dex induced significant down-regulation of GR, co-treatment of TPCA-1 significantly impaired the down-regulation. To validate that the effect of TPCA-1 on GR down-regulation is due to the inhibition on IKK2, we examined the effect of IKK2 knockdown using siRNAs. Compared to cells transfected with non-targeting RNA oligos, both basal level of GR and GR level upon Dex treatment increased in cells transfected with IKK2 siRNA (Fig. 4E). Taken together, these results indicate that inhibition of IKK2 attenuates Dex-induced GR down-regulation, leading to an increase in both the protein level and the activity of GR.

## Discussion

GCs regulate the expression of pro-inflammatory genes and are widely used as therapy for inflammatory diseases, but are hampered by severe side effects and poor responses in some patients. We have performed a high-throughput screening and identified multiple chemical inhibitors and enhancers that modulate the anti-inflammatory function of GR. Our work not only contributes to a better understanding of how GR function is regulated, but also may lead to the development of novel drugs that improve GC-based therapy.

We identified and characterized two IKK2 inhibitors as GR enhancers. Our findings demonstrated synergistic effects of IKK2 inhibitors on GC-mediated NF-*κ*B repression. IKK2 inhibitors suppress NF-κB repression on their own and have been shown to suppress inflammation *in vitro* and in different models of inflammation diseases, including asthma and COPD^36^. Thus it would be of interest to further explore the combination of glucocorticoids and IKK2 inhibitors to reduce inflammation in asthma and other inflammation diseases. Biochemical characterization revealed an unexpected role of IKK2 in GR down-regulation. Consistent with a role of IKK2 in IKK complex phosphorylation and NF-κB activation, IKK2 inhibitors impair IL1β induced NF-κB activation. Importantly, low concentrations of the inhibitor synergistically potentiated NF-κB repression by GR, and TPCA-1 also enhanced transactivation by GR. The effects of IKK2 inhibitor on both GR trans-repression and trans-activation suggest that IKK2 regulates GR in a NF-κB independent manner. A major mechanism of IKK2 function is via phosphorylating its substrate and further promoting substrate degradation^38, 39^. Although IKK2 inhibitors did not alter GR phosphorylation at the known sites, it is still possible that IKK2 phosphorylates GR at an unidentified site(s) and thereby promotes GR degradation. In addition, we cannot also rule out the possibility that IKK2 may involve in the phosphorylation/degradation of other repressive GR factors. Further biochemical characterization is needed to elucidate the exact mechanism by which IKK2 regulates GR stability.

We identified two DNA intercalating chemicals of different structures, pyrromycin and camptothecin, as GR inhibitors. Pyrromycin is a member of anthracyclines that exhibit broad bioactivities^41^, Camptothecin is a DNA topoisomerase I inhibitor^32^. Both chemicals are reported to inhibit RNA transcription. The non-specific inhibition of transcription may contribute to inhibition of GR transactivation by these chemicals. However, at low concentrations, these chemicals did not substantially alter NF-κB activation. Thus, non-specific transcription inhibition is less likely to be the mechanism accounting for the observed inhibitory effects on NF-κB repression. Rather, intercalation of chemicals with DNA may allow specific interactions between NF-κB, GR and chromatin that lead to the impairment of tethered trans-repression by GR. Given the use of camptothecin analogues in cancer therapy^32^ and the wide therapeutic application of glucocorticoids, the identification of camptothecin as a GR inhibitor raises the question of whether camptothecin and it analogues diminish the anti-inflammatory effects of glucocorticoids in chemotherapy patients. The systemic effect of camptothecin and its analogues on glucocorticoids thus may warrant further investigation.

Our screen identified two GSK3β specific kinase inhibitors and a non-specific kinase inhibitor, which also targets GSK3β, as GR inhibitors. GSK3β inhibition has been shown to suppress GR trans-activation^29^. Together, these results support a positive role of GSK3β in GR activation. However, it is also reported that GSK3β-mediated GR phosphorylation at serine 404 inhibited GR dependent NF-κB trans-repression^42^. Additional studies are needed to further clarify the role of GSK3β in GR regulation. Furthermore, it may be worthwhile to test whether GSK3β activation enhances GR function.

Ideally GR activators would enhance only tethered trans-repression but not trans-activation, which is associated with side effects. Further screening of a larger chemical compound library could identify more compounds that specifically improve the tethered trans-repression activity of GR. An alternative approach may be to develop more specific screening methods. For example, GR monomers are thought to control tethered repression, whereas GR dimers stimulate trans-activation^43, 44^. Thus, a screen designed to identify GR dimerization inhibitors may select chemicals that specifically potentiate anti-inflammation activity of GR and diminish the GC-related side effects. A multiplexed reporter screening assay has been developed to search target gene specific GR transactivation inhibitors^21^. Modification of that method may allow the assessment of tethered repression and trans-activation in a single screen. Such a screen could identify more potent and specific GR activators that will improve current GC-based therapies for inflammatory lung diseases.

## Methods

### Cell culture

A549 human lung tumor epithelial cells and primary human airway smooth muscle (HASM) cells were maintained in high-glucose DMEM medium containing 10% FBS. For luciferase and IL-6 assays, A549 cells were grown in DMEM/F12(1:1) medium without red dye(Life), supplemented with 3% dialyzed FBS (Life), glutamine, penicillin and streptomycin. Stable A549 cells were grown in the presence of 3% dialyzed serum to minimize the interference of endogenous glucocorticoids in the serum. To measure IL6 release by HASM cells, cells were grown in high glucose DMEM medium supplemented with glutamine, penicillin and streptomycin.

### Plasmids and transfections

pGL4.32[*luc2P*/NF-κB-RE/Hygro] was obtained from Promega. Luciferase reporter plasmid pGILZ-luc were gifts from Anthony Gerber (University of Colorado, Denver). For establishment of the A549/NF-κB-luc reporter cells, plasmid pGL4.32[*luc2P*/NF-κB-RE/Hygro] was linearized and transfected into A549 cells with TurboFect transfection reagent (Thermo). Transfected cells were selected for stable transgene expression by co-culture with 0.4 mg/mL Hygromycin (Life). Individual hygromycin-resistant colonies were isolated, expanded in culture and characterized for transgene expression by luciferase assay. For transient transfection, A549 cells were transfected using Fugene 6 reagent (Roche).

### SiRNA transfection

Transfection of siRNA was done using DharmaFECT 1 reagent (Thermo Scientific). Non-targeting control siRNA and IKK2-specific siRNA (sense 5′-cau uua ccu ggc aug aga a(dT)(dT)-3′, antisense 5′-uuc uca ugc cag gua aau g(dT)(dT)-3′) were purchased from Sigma-Aldrich (Mission siRNA; St. Louis, MO, USA).

### Luciferase assays

Luciferase assays were performed using Steady-Glo luciferase agent (Promega). Luciferase activity (immunofluorescence) was measured by using Envision 3 (Perkin Elmer) or Synergy 2 plate reader (BioTek).

### IL-6 assay

IL-6 release in cell culture medium was measured using the Quantikine ELISA kit (R&D Systems).

### Compound screen

We developed and optimized an automated, high-throughput screen to identify potential GR chemical modulators present in a small molecule library of 8,148 bioactive compounds.The screen was conducted at the Institute of Chemistry and Cell Biology-Longwood at Harvard School of Medicine. The known bioactive collections, including 8148 of natural products and synthetic compounds (http://iccb.med.harvard.edu/libraries/compound-libraries/) were screened. This chemical library includes 640 FDA approved drugs, 731 molecules from NIH clinical collections that were previously used in human clinical trials, 899 synthesized kinase inhibitors and >5,000 additional commercial bioactive compounds, all of which are selected to maximize chemical structure and biological pathway diversity. For screening, A549/NF-κB-luc reporter cells were seeded into two 384-well white plates (Corning) at 4000 cells per well in 30 μL of DMEM/F12(1:1) medium without red dye, supplemented with 3% dialyzed FBS. The next day, 33 nL of library compounds in DMSO was pin transferred into individual wells by using a Epson robot (one compound per well). One hour later, either 5 ng/mL IL1β (Assay I) or 5 ng/mL IL1β + 2.5 nM Dex (Assay II) were added to one of the two plates, respectively. The final volume of assay was 35 μl/well. After 18 h of the treatment, 10 μL Steady-Glo luciferase agent plus 5 μl PBS were added to both plates, and the luciferase assay was performed. The Z factor of the assay is defined as 1–3(σ_*p*_ + σ_*n*_)/(μ_*p*_ − μ_*n*_), whereas the means (μ) and standard deviations (σ) of the both positive(p) and negative control(n) are given (ref. 25).

The assay conditions and procedures were optimized such that the Z factors for both assays were greater than 0.6.

The compounds were screened in duplicate plates to determine the median absolute deviation (MAD)-based Z score (Z’), which is defined as (Luc reading of compound-median Luc reading)/MAD. Compounds with Z’II > 3, DMSO% I in the range of 0.8–1 and II/I ratio 50% higher than DMSO control were considered to be GR specific inhibitors; compounds with Z’II < −3, II/I ratio 20% lower than DMSO control were identified as activators.

### Quantitative PCR (qPCR) analysis

Total RNA was isolated from cells by using QIAshredder and RNeasy kits (Qiagen). Oligo(dT)-primed cDNA was prepared from 200 ng of total RNA using SuperScript III First-strand Synthesis System (Invitrogen). qPCR reactions were performed using QuantiTect SYBR Green PCR kit (Qiagen) and included 0.5 μM primers. qPCR assay was performed using a StepOne Plus real time PCR machine (Applied Biosystems) and amplicon expression was analyzed using the ddCt method, normalizing to *β-actin* expression as an internal control.

### Immunoblot analysis

Cells were washed with PBS and lysed in Trion X-100 lysis buffer (50 mM Tris-HCl pH7.5, 150 mM NaCl, 1% Trion X-100) supplemented with protease inhibitor cocktail (Roche) and phosphatase inhibitor cocktail (Roche). Cell lysates were separated on NuPAGE 4–12% Bis-Tris gels (Invitrogen) and transferred to PVDF membranes (Bio-Rad). Immunoblot signals were developed using SuperSignal West Pico Chemiluminescent Substrates (Pierce Biotechnology). Primary antibodies used in the study included anti-GR antibody (Santa Cruz), anti-GR(Phospho-Ser203) antibody (Assaybiotech), anti-GR(Phospho-Ser211) antibody (Cell signaling), anti-GR(Phospho-Ser226) antibody (Assaybiotech), anti-IKK2 antibody (Cell signaling), anti-histone H3 (Santa Cruz), anti-GAPDH (GeneTex), and anti β-actin antibody (Santa Cruz).

### Cell fractionation

Cells were harvested and washed with PBS. The cytoplasmic and nuclear fractions were prepared by using NE-PER Nuclear and Cytoplasmic Extraction Reagents (Thermo).

### Statistical analysis

The significance of differences between two groups was evaluated using Student’s *t*-test (*P < 0.05; **P < 0.01; ***P < 0.005). All quantitative experiments are performed in triplicate samples. Data are expressed as mean ± SEM.

## Electronic supplementary material

Supplementary information
