# Supplementary material for: A high-throughput chemical screen identifies novel inhibitors and enhancers of anti-inflammatory functions of the glucocorticoid receptor
Source: Sci Rep. 2017 Aug 7;7:7405. doi: 10.1038/s41598-017-07565-2 (PMC5547123; doi:10.1038/s41598-017-07565-2)
Supplement: Supplementary file 1 — Supplementary information [file 41598_2017_7565_MOESM1_ESM.pdf]

Supplementary Files

A high-throughput chemical screen identifies novel inhibitors and enhancers of anti-inflammatory functions of the glucocorticoid receptor

Xiaofeng Jiang[1], Amber Dahlin[2], Scott T. Weiss[2], Kelan Tantisira[2] and Quan Lu[1]*

Supplementary Table 1: Bioactivities and assay II/I ratios of GR inhibitors and enhancers.

Compounds	Assay II/I ratio	Bioactivities
Alsterpaullone	0.70	GSK3β, Cdk1/cylin B inhibitor
RO0275062	0.85	GSK3β inhibitor
RO0317753	0.91	GSK3β inhibitor
Pyrromycin	0.77	DNA intercalation
Camptothecin	1.0	DNA topoisomerase I inhibitor
TPCA-1	0.28	IKK2 inhibitor
IKK2 inhibitor VI	0.30	IKK2 inhibitor

* Assay II/I ratio for DMSO is 0.4

Supplementary Figure S1: Effects of IL1β on NFκB activation in A549 cells (A), on IL-6 release in A549 cells (B), and on IL-6 release in human airway smooth muscle cells (C).

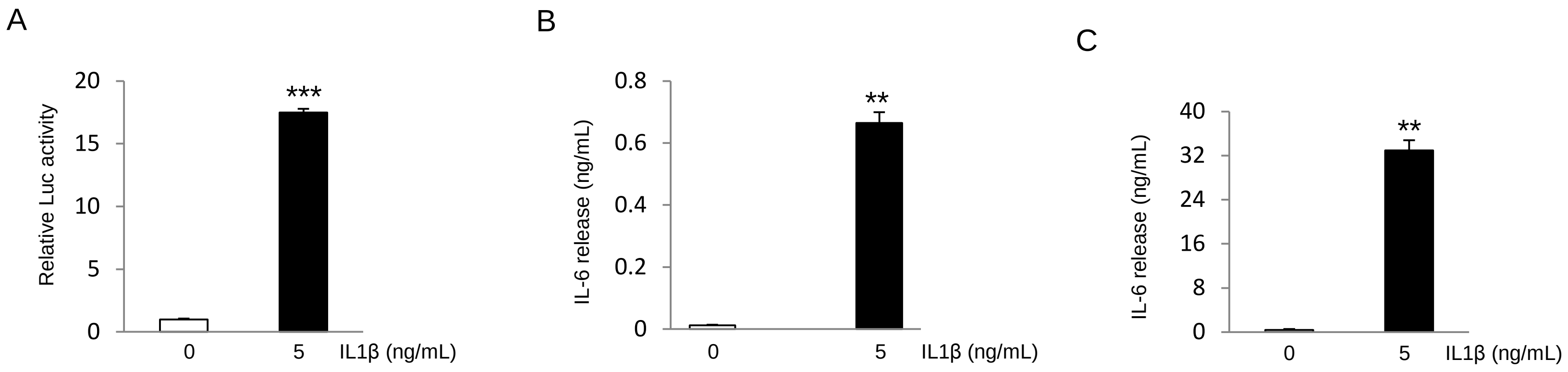

Supplementary Figure S2: Effects of TPCA-1 on GR mRNA expression. A549 cells were treated with different concentrations of TPCA-1 for 48 h, and qPCR was used to measure GR mRNA level.

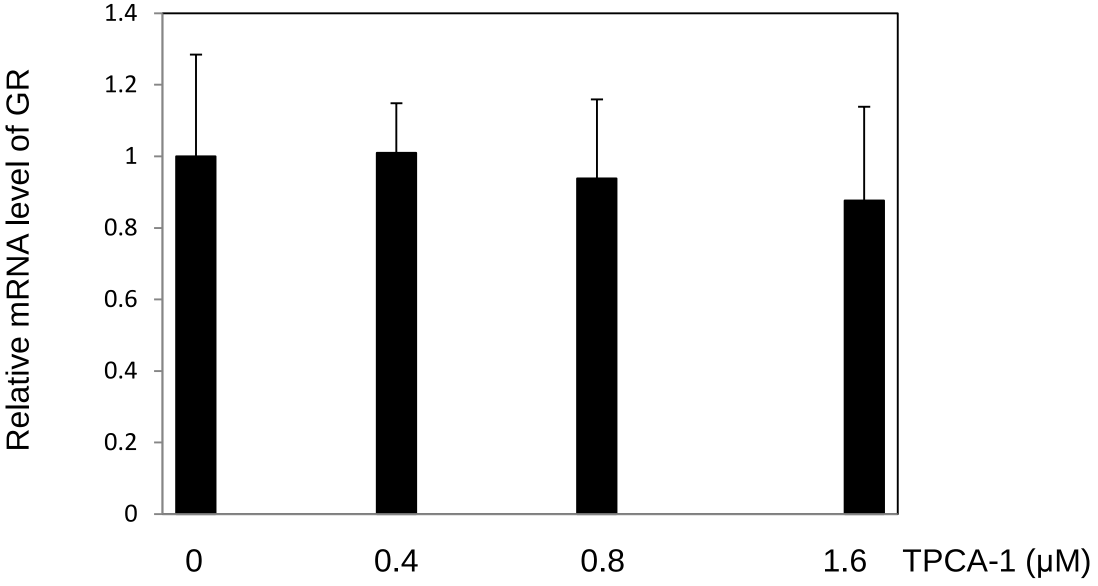

Supplementary Figure S3: *A*: Effect of IL-1β (5 ng/mL) treatment on NF-κB activation. *B*: Effect of TPCA-1 (0.8 μM) on NF-κB repression by Dex (0.5 nM) in A549/NF-κB-luc cells. All treatments were done for 4 h. Luciferase assays were performed after 4 h treatment.

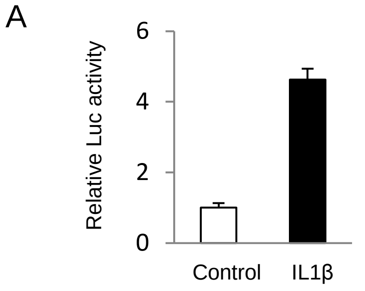

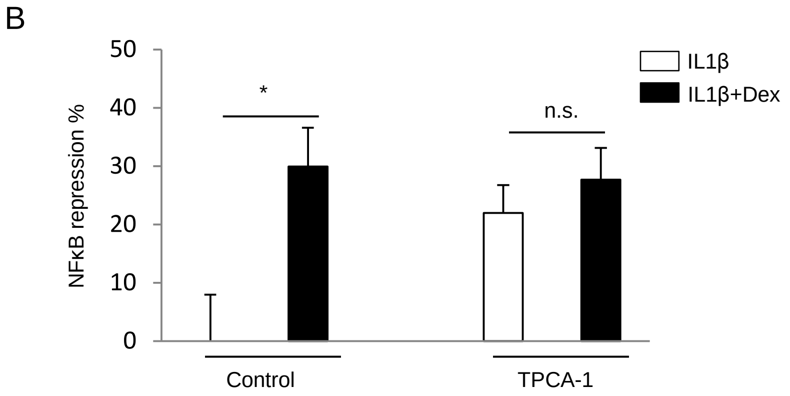

Supplementary Figure S4: GR antagonist RU486 attenuates the effect of Dex and TPCA-1 on NFκB repression. The reporter cells were treated with 5ng/mL IL-1β ± 0.5 nM Dex, 0.4 μM TPCA-1 or 5 nM RU486 as indicated for 18 h. Luciferase assays were performed, and NF-κB repression was calculated relative to the cells treated with IL1β. n.s., no significance. *, P <0.05. **, P < 0.01, ***P<0.005.

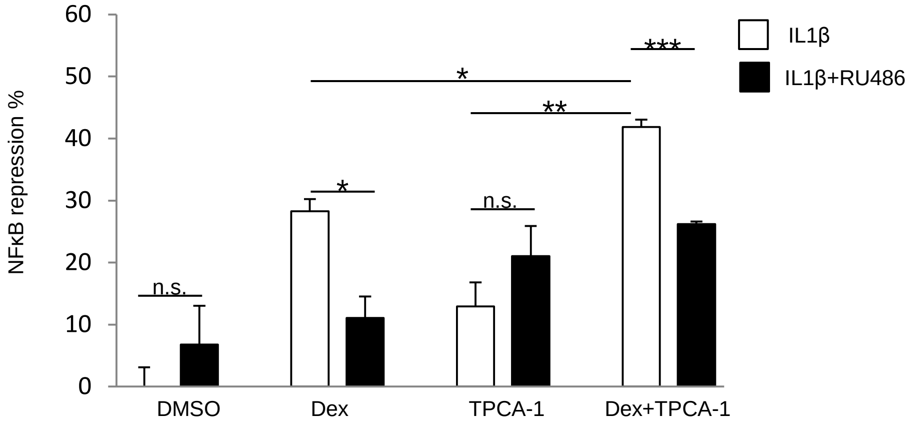

Supplementary Figure S5: Full-length blots for Fig.4

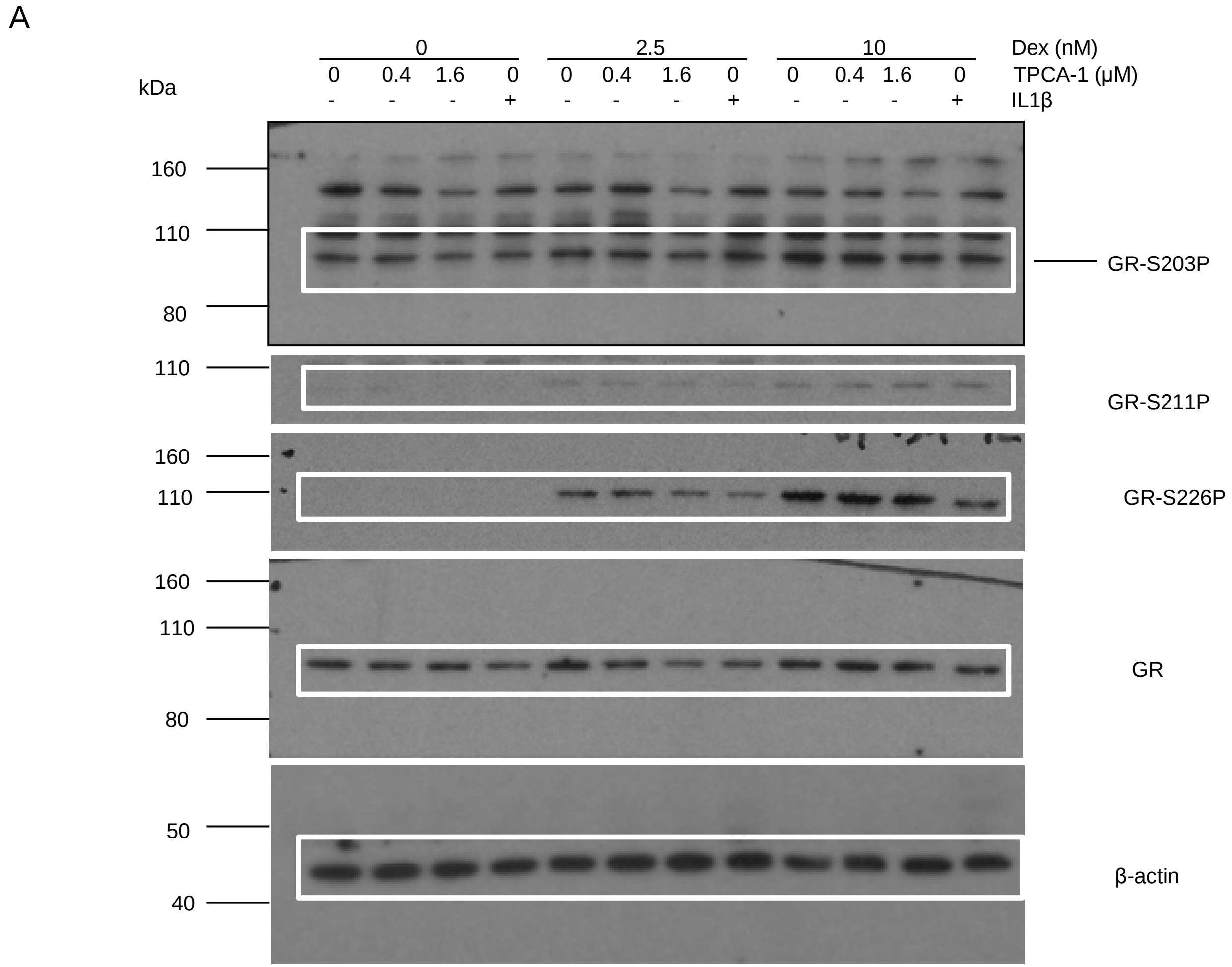

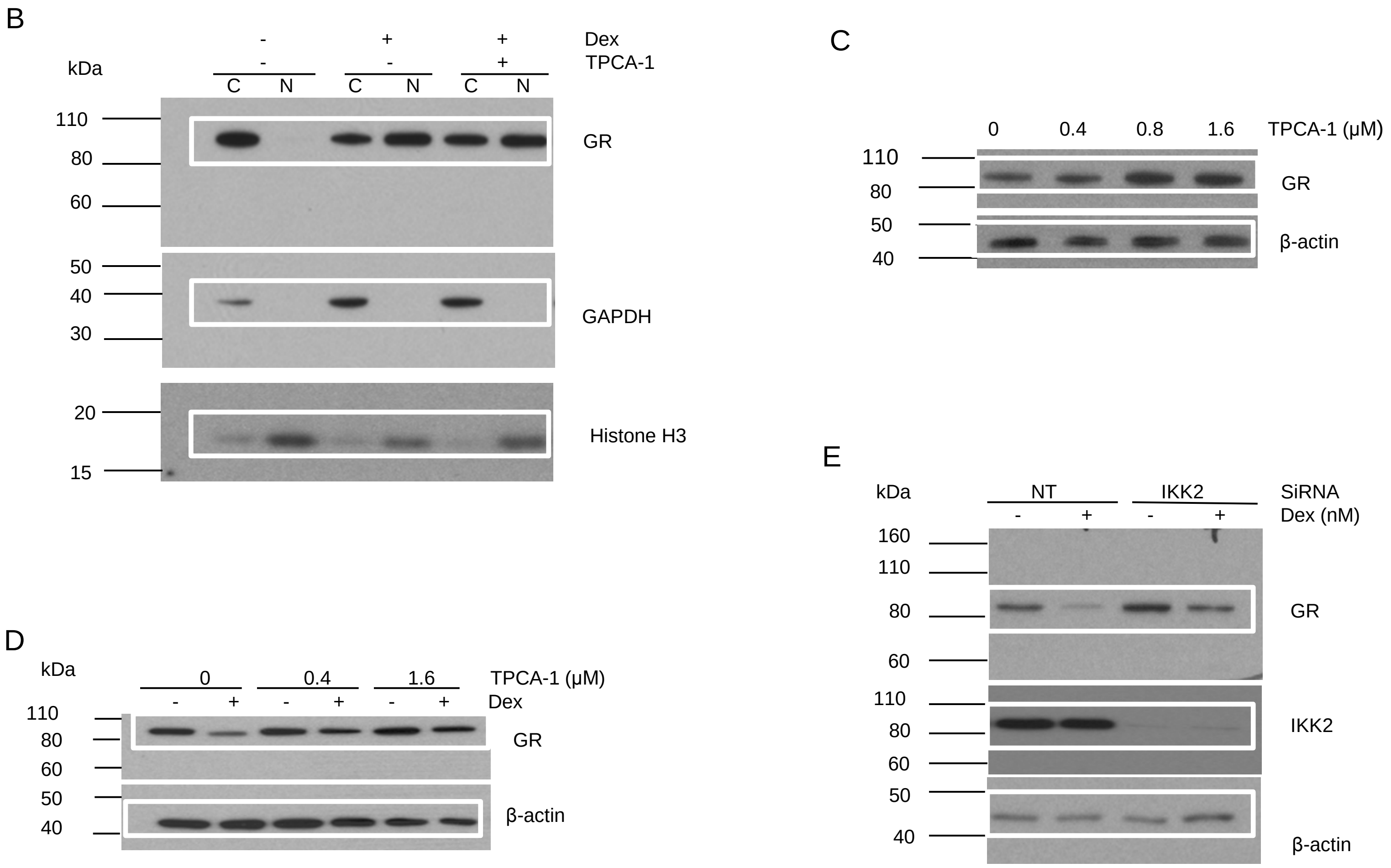
B
Dex
TPCA-1
kDa
C N C N C N
110
80
60
GR
50
40
30
GAPDH
20
15
Histone H3
C
0 0.4 0.8 1.6 TPCA-1 (μM)
110
80
GR
50
40
β-actin
D
kDa
0 0.4 1.6 TPCA-1 (μM)
- + - + - + Dex
110
80
60
50
40
GR
β-actin
E
kDa
NT IKK2 SiRNA
- + - + Dex (nM)
160
110
80
60
GR
110
80
60
IKK2
50
40
β-actin
